# Recurrent extramedullary plasmacytoma in asymptomatic multiple myeloma: a case report

**DOI:** 10.1186/s13256-014-0506-3

**Published:** 2015-02-19

**Authors:** Saskia EM Schols, Lidwine LW Tick

**Affiliations:** 1 Department of Hematology, Internal Medicine, Maastricht University Medical Center, Debyelaan 25, 6229 HX Maastricht, The Netherlands; 2 Department of Internal Medicine, Máxima Medical Centre, De Run 4600, 5504 DB Veldhoven, The Netherlands

**Keywords:** Extramedullary plasmacytoma, Multiple myeloma, p53 and MIB-1 expression

## Abstract

**Introduction:**

The gross majority of extramedullary plasmacytomas arise in the lymphatic tissue of the upper respiratory tract. On average, one third of patients with a solid plasmacytoma will develop multiple myeloma, resulting in a worse clinical outcome. We describe a case of rapid recurrent extramedullary plasmacytomas in the background of an asymptomatic multiple myeloma.

**Case presentation:**

A 71-year-old, white Caucasian woman presented with three extramedullary plasmacytomas occurring within a short time period. The third plasmacytoma was accompanied by progressive cervical pain and swallow dysfunction. Additional immunostaining test results were negative for CD56 and showed high MIB-1 expression in the extramedullary plasmacytoma and low MIB-1 expression in the bone marrow. A conventional swallow X-ray did not show any obstruction, however a magnetic resonance imaging scan of her cervical backbone revealed an extramedullary plasmacytoma, threatening her spinal cord. A short course of radiation therapy alleviated her pain and during almost a two-year follow-up period, the multiple myeloma remained asymptomatic, despite the rise in immunoglobulin A lambda levels. After the appearance of the third plasmacytoma, systemic chemotherapy was started to prevent the development of a fourth plasmacytoma, despite the asymptomatic character of the multiple myeloma.

**Conclusions:**

In this case report we describe the rapid appearance of extramedullary plasmacytomas in the background of an asymptomatic multiple myeloma. An immunohistochemical analysis was negative for CD56 and showed high MIB-1 expression in the extramedullary plasmacytoma and low MIB-1 expression in the bone marrow, contributing to the potential underlying pathophysiology of the recurrent extramedullary plasmacytomas and their genetic changes. Systemic chemotherapy was started and no fourth extramedullary plasmacytoma has developed since.

**Electronic supplementary material:**

The online version of this article (doi:10.1186/s13256-014-0506-3) contains supplementary material, which is available to authorized users.

## Introduction

Multiple myeloma is a neoplasm of proliferating monoclonal B cells which are terminally differentiated (plasma cells). The process of the development of mature B cells into a malignant plasma cell population is a multi-step process [1]. During the initial malignant transformation, the plasma cells resides in the bone marrow. However, later on in the transformation, extramedullary manifestations can occur such as the development of plasmacytomas [2].

Plasmacytomas are included in the World Health Organization (WHO) classification and are associated with the production of a monoclonal immunoglobulin or light chains [3]. In the absence of systemic involvement, the WHO describes two types of plasmacytomas: solitary plasmacytoma of the bone and extramedullary plasmacytoma (EMP). More than 80% of EMPs arise in the rich lymphatic tissue of the upper respiratory tract. In 17 to 33% of plasmacytomas, the extramedullary disease will develop into multiple myeloma.

The mean age of developing extramedullary plasmacytoma is 55 years, with a predominance for the female sex. Solitary EMPs are highly radiosensitive and many case reports have shown good disease control with external beam radiation therapy, with a 10-year overall survival rate of 70% [4]. Here, we present a case of recurrent EMPs in the upper part of the body in a woman with an asymptomatic multiple myeloma without signs of organ involvement.

## Case presentation

A 71-year-old white Caucasian woman was diagnosed with an immunoglobulin A (IgA) lambda multiple myeloma stage I in June 2011. At this time, she presented to the outward clinic of the Máxima Medical Centre Veldhoven with a painless soft tissue swelling near her right sternoclavicular joint. An immunocytological examination of this swelling showed an IgA lambda monoclonal plasma cell population analogical to an EMP. Further immunostaining tests were negative for CD56, and revealed a high MIB-1 index.

Additional blood tests revealed normal kidney function, no anaemia, a normal calcium level, no quantifiable M-protein and no lytic bone lesions. A bone marrow biopsy showed a monoclonal IgA lambda plasma cell population (10 to 20% presence) and she was treated with local radiation therapy.

One year later, she returned to the outward clinic with a swelling on her chest which was increasing in size. A magnetic resonance imaging (MRI) scan of her thorax showed a soft tissue pre-sternal swelling, suspected to be an EMP. As with the first EMP, she was treated with radiation therapy.

Two months after the development of her second EMP, she noticed some difficulty with swallowing and she experienced localised cervical pain. In the next few weeks, her ability to swallow deteriorated and eventually she could no longer eat solid food and started to lose weight. Her cervical pain increased with movement of her neck and was accompanied by tingling in both arms and hands. There was no loss of muscle power.

**Figure 1:**
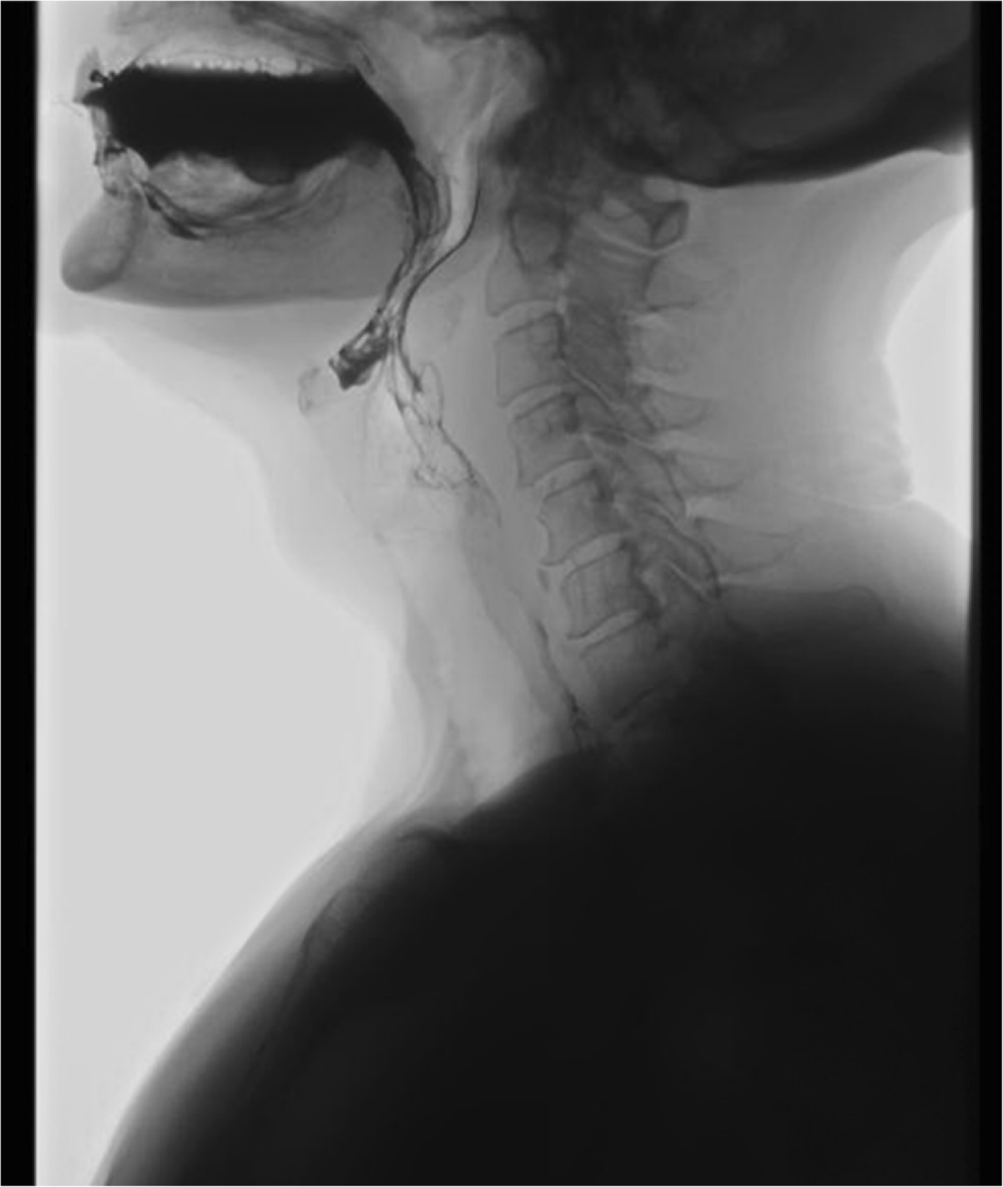
**Swallow X-ray with oral contrast.** No marked obstruction was visible during the swallow act.

**Figure 2:**
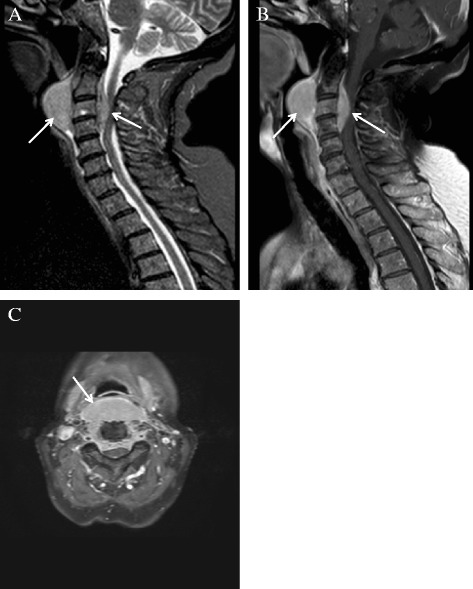
**Cervical backbone magnetic resonance images showing a solid tumor of soft tissue extending from the spinal cord (arrows). A** and **B** represent sagittal images, **C** represents the axial image.

At her presentation to the outpatient service, she was very anxious and was experiencing fear of suffocating. She had an extensive list of pain medication, however her pain was able to be brought under control. Her additional physical examination showed tense cervical muscles, no palpable swelling of her throat and no evidence of neurological loss. Her blood examination showed a hemoglobin level of 7.4mmol/L, a creatinine level of 62umol/L, a calcium level of 2.24mmol/L and an increased IgA lambda of 17.3g/L (compared to 6.0g/L at her diagnosis in 2011, Table 1). A swallow X-ray with oral contrast did not show any dysfunction or limitations in her intrapharyngeal lumen (Figure 1). In addition, an MRI scan of her cervical backbone was performed, which revealed a solid tumor of soft tissue at paravertebral level C2-3 with extension to the spinal cord, which we suspected to be a plasmacytoma (Figure 2). Her bone marrow biopsy showed an increase in her monoclonal plasma cell population to 34%. Additional immunostaining tests showed a low MIB-1 index and were negative for CD56.

**Table 1:** **Biochemical parameters at diagnosis and after one year at the time of recurrence of her third extramedullary plasmacytoma**

**Parameter**	**May 2011**	**June 2012**
Hemoglobin (7.5-10mmol/L)	8.9	7.4
Leucocytes (4–10 × 109/L)	5.7	10.2
Creatinine (49-90umol/L)	74	62
Calcium (2.15-2.55mmol/L)	2.24	2.16
ß2-microglobulin (800-2200mg/L)	2400	2450
Total IgA lambda g/L	6.0	17.3

**Figure 3:**
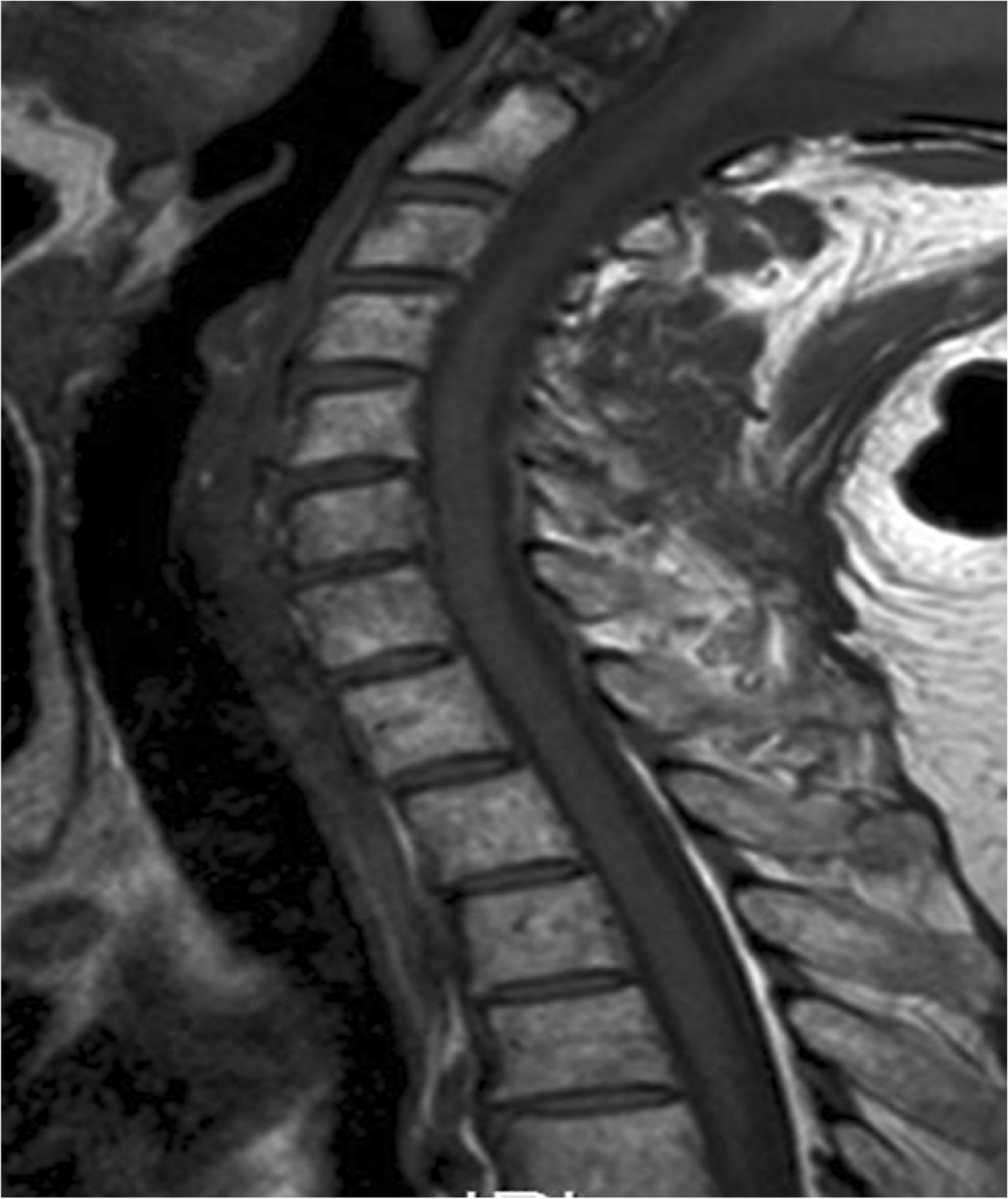
**Cervical backbone magnetic resonance image after irradiation therapy and adjunctive chemotherapy.** Her spinal cord compression is shown to have disappeared and the intrapharengeal lumen is free of plasmacytoma.

In view of the potential threat to her spinal cord, localised radiotherapy was started directly. This consisted of five sequences at 20Gy. After only two radiation sessions, the pain was relieved and she could swallow liquid food again and needed no additional pain medication. Because of the rapid recurrence of three plasmacytomas in one year, systemic chemotherapy was started in the form of melphalan, prednisone and thalidomide. After three cycles of chemotherapy she continued to have a satisfactory response (Figure 3).

## Discussion

The presence of an EMP at the time of diagnosing multiple myeloma is associated with a poor outcome compared to cases in which plasmacytomas develop during the course of the disease (median overall survival 28 versus 68 months) [2]. In a recent study by Varettoni *et al*., the incidence and outcome of EMP was described in a large cohort study of patients with multiple myeloma [5], comparing three distinct time periods; 1971 to 1993, 1994 to 1999 and 2000 to 2007. Of the 1,003 patients who were studied, only 13% had EMPs (7% at the time of diagnosis and 6% during the follow-up period). Interestingly, the incidence of EMP seems to have increased over recent years. One explanation for this could be the more sensitive imaging techniques that are used today, such as MRI scanning. Concerning our case report, a conventional swallow X-ray did not reveal the cause of the swallow dysfunction. However, her MRI scan showed the EMP in the lower part of her pharynx.

Another important factor for the increased incidence in EMPs is prolonged survival due to new treatment strategies, such as the combination of radiotherapy with novel agents like proteasome inhibitors and immune modulators [6,7]. EMPs are highly radiosensitive; more than 80% of patients can achieve local control [8]. However, in the presence of rapid recurrence of plasmacytoma with evidence of an increase in the monoclonal component of protein electrophoresis, chemotherapy after radiotherapy may be considered [9]. The precise role of new therapeutic agents in the management of plasmacytomas has yet to be determined.

EMPs usually arise in the submucosa of the upper respiratory tract and the gastro-intestinal tract, as in the last plasmacytoma of this case report. However, they can arise in any part of the body [9]. For choosing the right treatment option (local radiotherapy versus systemic chemotherapy), systemic involvement of multiple myeloma must be ruled out. There are several criteria that must be confirmed in order to diagnose the presence of an EMP without symptomatic multiple myeloma [10], which are as follows: normal levels of hemoglobin, calcium and kidney function, an absence of lytic bone lesions, a low serum M-protein level and a tissue biopsy showing a monoclonal plasma cell population.

**Figure 4:**
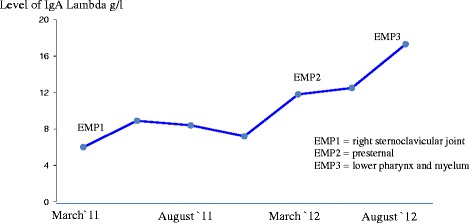
**Level of immunoglobulin A (IgA) lambda during the follow-up period after diagnosing multiple myeloma and the first extramedullary plasmacytoma (EMP).** Additionally, the development of the three plasmacytomas are shown in association with the time of complaints.

Concerning our patient, there was no evidence of anaemia, hypercalcemia, kidney dysfunction or lytic bone lesions. Her M-protein level did rise from 6.0 to 17.3g/L (Figure 4). Although this is nearly a threefold increase, it was not accompanied by any evidence of organ involvement.

The pathophysiological mechanism for EMPs is not yet fully determined. One suggestion is that multiple myeloma cells and the bone marrow microenvironment tightly interact with each other. This is mediated by multiple soluble factors and adhesion molecules. Integrins acting as receptors on stromal cells and the extracellular matrix play an important role in the homing of multiple myeloma cells in the bone marrow [11]. Changes in these adhesion molecules and integrins can lead to the migration of multiple myeloma cells to the bloodstream (plasma cell escape), and finally to the development of an extra-osseous plasmacytoma. In addition, for plasma cells to migrate outside of the bone marrow, genetic changes must occur which give these migrating plasma cells their aggressive nature. Recent studies have investigated the expression of several (glyco)proteins in multiple myeloma [12-14]. Mutations in genes encoding these proteins, like p53, play a major role in the aggressive character of the disease. p53 is a nuclear protein which provides genomic stability by regulating the cell cycle. Another protein, CD56 is usually present as a membrane glycoprotein on natural killer cells and T cells and is involved in cell adhesion and migration. CD56 is expressed in more than 70% of plasma cells in multiple myeloma, however it is not expressed in normal plasma cells. There is evidence that CD56 is downregulated in extramedullary multiple myeloma.

A recent study by Sheth *et al*. investigated the association of p53, CD56 and MIB-1 expression in intramedullary and extramedullary biopsies of multiple myeloma patients with progression to extramedullary myeloma [15]. The results showed that in extramedullary multiple myeloma biopsies, nuclear p53 immunoreactivity and the MIB-1 staining index were significantly higher than in intramedullary and EMP biopsies. CD56 staining was expressed in a low number of all biopsies. To conclude, the study demonstrated an association of high p53 immunoreactivity in extramedullary multiple myeloma and an increased cell proliferation (MIB-1 staining), probably due to progression of the disease. Concerning our patient, there was a high expression of MIB-1 and negative CD56 level in the EMP. On the contrary, her bone marrow biopsy showed a low MIB-1 expression. Unfortunately we have no information about the nuclear p53 level. However the high MIB-1 staining in the EMP points to the aggressive character of the disease. Furthermore, downregulation of CD56, as in both our biopsies, is associated with more aggressive disease progression by the facilitated extravasation of myeloma cells [13,15]. More studies are needed to determine the exact association between p53, CD56 and MIB-1 expression and progression of multiple myeloma and EMP.

In conclusion, the increased availability of performing immunohistochemical staining allows the physician to detect more aggressive EMPs, and thus to start systemic chemotherapy sooner. For future management this will implicate more targeted therapies that interfere with the cell proliferation function of p53 and MIB-1 expression.

## Conclusions

In this report, we present a case of aggressive development of EMPs in the background of an asymptomatic multiple myeloma. Despite an increase in the IgA lambda level, no organ involvement occurred. Few studies have been performed considering the implication of genetic changes such as nuclear accumulation of p53 and MIB-1 expression on the aggressive nature of multiple myeloma and EMPs. There seems to be an important association between accumulation of p53, a high MIB-1 expression and downregulation of CD56 with the progression of multiple myeloma and the presence of plasmacytomas. In our patient, the first EMP exposed a high level of MIB-1 and was negative for CD56, with concomitant low MIB-1 expression and no evidence of CD56 in the bone marrow. These genetic changes pointed to the aggressive nature of the plasmacytoma and justify the start of systemic chemotherapy after the appearance of the third plasmacytoma.

## Consent

Written informed consent was obtained from the patient for publication of this case report and any accompanying images. A copy of the written consent is available for review by the Editor-in-Chief of this journal.

## Abbreviations

EMP: Extramedullary plasmacytoma
Gy: Gray
IgA: Immunoglobulin A
M-protein: Monoclonal protein
MRI: Magnetic resonance imaging
